# Clinical Analysis of Five Cases of AIDS-related Non-Hodgkin Lymphoma

**DOI:** 10.12669/pjms.326.10172

**Published:** 2016

**Authors:** Shubo Zuo, Na Xu, Zhongkun Li, Na Li, Hong Xia, Hongtao Ren, Huizheng Bao

**Affiliations:** 1 Shubo Zuo, MD, Department of Medical Oncology, Jilin Tumour Hospital, Changchun 130000, Jilin Province, PR China; 2 Na Xu, MD, Department of Medical Oncology, Jilin Tumour Hospital, Changchun 130000, Jilin Province, PR China; 3 Zhongkun Li, MD, Department of Medical Oncology, Jilin Tumour Hospital, Changchun 130000, Jilin Province, PR China; 4 Na Li, MD, Department of Medical Oncology, Jilin Tumour Hospital, Changchun 130000, Jilin Province, PR China; 5 Hong Xia, MD, Department of Medical Oncology, Jilin Tumour Hospital, Changchun 130000, Jilin Province, PR China; 6 Hongtao Ren, MD, Department of Medical Oncology, Jilin Tumour Hospital, Changchun 130000, Jilin Province, PR China; 7 Huizheng Bao, MD, Department of Medical Oncology, Jilin Tumour Hospital, Changchun 130000, Jilin Province, PR China

**Keywords:** Antiretroviral therapy, Chemotherapy, Human immunodeficiency virus/acquired immunodeficiency syndrome, Non-Hodgkin lymphoma

## Abstract

**Objective:**

Secondary malignancy is a major life-threatening complication facing patients afflicted with acquired immunodeficiency syndrome (AIDS). This study aimed to retrospectively review clinical features and treatment course of five patients with AIDS-associated non-Hodgkin lymphoma (A-NHL) in Jilin Tumor Hospital.

**Methods:**

Five A-NHL patients were retrospectively and consecutively hospitalized at our oncological unit between January 2012 and June 2014. All patients received pre-emptive highly active antiretroviral therapy (HAART) and chemotherapy, and were subsequently followed up at the outpatient clinic. All five patients were male, aged 27–53 years, and afflicted with A-NHL involving upper jaw, right inguinal region, right-side gingiva, mediastinum, or right-side neck. Histology showed diffuse large B-cell lymphoma (n = 3) or plasmablastic lymphoma (n = 2).

**Results:**

Two patients achieved complete remission after HAART and chemotherapy, whereas other three patients required a second-line treatment, with two achieving stable disease and one dying within a follow-up period of 0.5−2 years.

**Conclusion:**

The findings of the present study showed that A-NHL is a disease often diagnosed in the middle-to-late stages, with diverse clinical manifestations and short overall survival. In the cases reviewed in this study, HAART in combination with standard dose or high-dose chemotherapy, HAART and molecular targeted chemotherapy was administered, and these treatments proved to be effective for improving the prognosis of these patients. Moreover, the CD4+ cell count was important for determining the prognosis of patients.

## INTRODUCTION

Human immunodeficiency virus (HIV) specifically infects CD4^+^ T lymphocytes and destroys immune surveillance within the human body. In addition to life-threatening opportunistic infections, rare malignancies can occur in patients afflicted with acquired immunodeficiency syndrome (AIDS), including Kaposi carcinosarcoma, non-Hodgkin lymphoma (NHL), cervical cancer, Hodgkin’s Lymphoma, lung cancer, laryngeal cancer, colorectal cancer, breast cancer, prostate cancer, and testicular cancer.^1^,^2^ AIDS patients with complicating secondary malignancies usually have a very poor prognosis.^3^

AIDS-associated NHL (A-NHL) is one of the most common AIDS-associated malignancies.^4^ Like NHL in the general population, A-NHL exhibits a highly variable profile in clinical and histologic characteristics, with B-cell lymphoma, especially diffuse large B-cell lymphoma, as the most frequent subtype, accounting for more than 90% of all A-NHL cases.^5^,^6^ NHL as the primary manifestation of AIDS is encountered less frequently in oncologic practice. This study aimed to retrospectively review the clinical features and treatment courses of five patients with A-NHL treated in a tertiary care in Jilin Tumor Hospital.

## METHODS

The study protocol was approved by the Institutional Review Board of local Hospital. Medical charts of NHL patients consecutively hospitalized from January 2012 to June 2014 were retrospectively reviewed to identify patients with complicating AIDS. The eligibility criteria were as follows: having a histologic and/or immunocytogentic diagnosis of NHL at the initial visit; with a confirmed diagnosis of AIDS; and have an evaluable inpatient and follow-up records. All patients provided informed consent in writing before receiving any invasive diagnostic and/or therapeutic interventions.

Contrast-enhanced computed tomography (CT) and plain magnetic resonance imaging (MRI) scans were performed on the chest, abdomen and pelvis as routine; positron emission tomography/CT scanning was conducted if the economic situation of the patient allowed.^7^ The lactate dehydrogenase assay and bone marrow aspiration and biopsy were also done in all patients. Histology and immunohistochemistry of the biopsy specimen were reviewed and staged by a centralized pathologic unit in accordance with *the 2008 World Health Organization (WHO) Classification of Lymphoma*^8^ and the Ann Arbor staging system,^9^ respectively. Lumbar puncture was done after the diagnosis of NHL was confirmed. Patients showing cerebrospinal involvement underwent additional contrast-enhanced head MRI scanning with the plain spin echo sequence.

An HIV-1 antibody test was ordered in all NHL patients prior to administration of chemotherapy along with virologic and/or serologic tests for other common infectious diseases, such as hepatitis B/C viruses, human papilloma virus, and cytomegavirus. T-cell subtype analysis was done in a patient with positivity to HIV-1 antibody. HIV infection was confirmed by the Jilin Provincial Center for Disease Control (CDC) according to *The Diagnostic Standards and Treatment Principles for HIV/AIDS* established by the Chinese Medical Association^10^ with reference to the WHO recommendation^11^ and the United States CDC guidelines.^12^

## RESULTS

Out of approximately 100 NHL patients hospitalized during the study period, five patients had AIDS. The five patients were male and had a median age of 28 years (range, 27−53 years). The clinical data for these A-NHL patients are shown in Table I. Their chief complaints included asthenia (n = 5), persistent fever (n = 3), and dramatic weight loss (n = 1). A review of their social history showed that all five patients were males having sex with males. All patients were seronegative for hepatitis virus B, hepatitis virus C, and Epstein-Barr virus. Comorbidities included syphilis (n = 2), polycystic kidney disease (n = 1), and azontemia/chronic renal failure (n = 1).

**Table-I:** Clinical data of A-NHL patients (n = 5).

No.	Age (Years)	Comorbidities	HAART		

			Duration before A-NHL (mo)	Scheme	CD4^+^ cell count (×10^6^/L)
1	49	Syphilis	3	3TC+AZT+NVP	95
2	27	None	9	3TC+D4T+EFV	294
3	53	PKD/CRF	3	3TC+NVP+AZT	110
4	28	None	12	3TC+AZT+EFV	30
5	28	Syphilis	2	3TC+AZT+EFV	135

3TC, lamivudine; A-NHL, AIDS-associated non-Hodgkin lymphoma; AZT, zidovudine; CRF, chronic kidney failure; D4T, stavudine; EFV, efavirenz; HAART, highly active antiretroviral treatment; NVP, nevirapine; PKD, polycystic kidney disease.

All patients had received highly active antiretroviral therapy for 2−12 months before seeking oncologic care, with an undetectable HIV RNA and a CD4^+^ cell count of 4−294×10^6^/L (reference limits 400−1,600×10^6^/L). The lymphomas involved the upper jaw, right inguinal region, right-side gingiva, mediastinum, and right-side neck in one case each. A patient suffering from right-side ptosis exhibited intracranial lymphoma infiltration on the head MRI scan, and a second patient complained of neurologic symptoms but showed no clinically significant abnormalities on head and thoracolumbar MRI scans. The remaining three patients had no neurologic symptoms or signs or positive results on cerebrospinal fluid (CSF) examination.

The pathology data for these cases are shown in Table II. Histologic examination showed diffuse large B-cell lymphoma (n = 3) or plasmablastic lymphoma (n = 2), with a normal (n = 3) or increased (n = 2) serum lactate dehydrogenase (LDH) level. Clinical staging was I (n = 1), II (n = 2), III (n = 1) or IV (n = 1). The international prognostic indexes (IPI scores) of the patients were between 0 and 2 points.

**Table-II:** Pathologic data of A-NHL patients (n = 5).

No.	Initial manifestation	sLDH	Pathology	Affected sites	Staging	IPI
1	Left palatal mass	Normal	PBL	Left upper gum, hard palate, maxillary bone	I	1
2	Right inguinal mass	Increased	DLBCL (N-GCB)	Left supraclavicularis, bilateral groins, abdominopelvic nodes	III	1
3	Right gingival mass	Increased	PBL	Parietal bone, occipital bone, submedial area, left mastoid wall, right maxillary bone, alveolar bone, and L5-S2	IV	2
4	Cough and fever	Normal	DLBCL	Left mediastinum and main bronchus	II	1
5	Right neck mass	Normal	DLBCL (GCB)	Both sides of the neck	II	0

A-NHL, AIDS-associated non-Hodgkin lymphoma; DLBCL, diffuse large B-cell lymphoma; GCB, germinal center B-cell like; IPI, International Prognosis Index; NGCB, non-germinal center B-cell like; PBL, plasmablastic lymphoma; sLDH, serum lactate dehydrogenase.

The treatment courses and emergent adverse effects are shown in Table-III. All five A-NHL patients received systemic chemotherapy under pre-emptive HAART. Four patients received additional rituximab therapy, and two patients received sequential radiotherapy. The chemotherapy regimens included EPOCH (n = 3), CVP+CHOP (n = 1), and CHOP (n = 1). Two patients achieved complete remission (CR) after HAART and chemotherapy. The other three experienced a partial response (PR) or disease progression and required a second-line treatment, with two achieving stable disease and one dying within a follow-up period of 0.5−2 years. The five patients included in this study all experienced the side effects of grade 3-4 myelosuppression and grade 1-2 gastrointestinal reactions. Zidovudine was switched to stavudine in two patients with grade 4 myelosuppression. CD4^+^ cell counts showed a progressive increase in three patients but no significant improvement in two patients (Table IV).

**Table-III:** Treatment and treatment-related side effects.

No.	CT	Response	MS	GI reaction	Sepsis	RT	2^nd^-line treatment
1	R-CHOP×6	PR	Grade 3-4	Grade 2	NA	Regional	MTX, TMZ, intrathecal
2	R-CHOP×6+prophylactic intrathecal	CR	Grade 2-3	Grade 1	NA	Regional	NA
3	R-CHOP×8	PR	Grade 4	Grade 2	NA	Regional	GEMOX
4	R-CHOP×4	PD	Grade 4	Grade 2	A. baumannii	Regional	Hyper-CVAD
5	CHOP×6	CR	Grade 4	Grade 1	NA	NA	NA

CHOP, cyclophosphamide + doxorubicin + vincristine + prednisone; CR, complete remission; CT, chemotherapy; GEMOX, gemcitabine + oxaliplatin; GI, gastrointestinal; Hyper-CVAD, cyclophosphamide + vincristine + dexamethasone followed by methotrexate + cytarabine; MTX, methotrexate; NA, not applicable; PD, disease progression; PR, partial remission; R-CHOP, rituximab + CHOP; RT, radiotherapy; TMZ, temozolomide.

**Table-IV:** Follow-up of CD4^+^ cell counts throughout treatment with HAART and chemotherapy.

Treatment and CD4^+^ T cell counts (×10^6^/L) on follow-up											

Case no.	Therapeutic method	Diagnosis	Months (mo) after diagnosis								
			3	6	9	12	15	18	21	24	27
1	3TC+AZT+NVP	95	146	183	201	246	268	298	350	361	372
2	3TC+D4T+EFV	294	298	280	247	207	200	230	275	284	302
3	3TC+NVP+AZT	110	142	150	181	191	200	245	260	252	254
4	3TC+AZT+EFV	30	4	0							
5	3TC+AZT+EFV	135	146	330							

Case No.-1 achieved a PR after six cycles of EPOCH (etoposide + prednisone + vincristine + cyclophosphamide + epirubicin) chemotherapy combined with four cycles of rituximab. Sequential radiotherapy was performed on the left alveolar bone, maxillary sinus, and ethmoid sinus and resulted in a CR. During the 1-year follow-up period, the patient complained of headache, dizziness, nausea, and vomiting; his CSF pressure was 300 mm H2O (80-180 mmH2O refernce limits), with a slightly increased CSF content of protein but without any clinically significant abnormalities on head MRI. Intrathecal monotherapies with methotrexate and temozolomide, two cycles of each, were given along with 50-mg cytarabine and 5-mg dexamethasone. His symptoms did not improve significantly, with newly-emerging tinnitus, weakness, and numbness of bilateral lower limbs and difficulty in defecation; head and spinal MRI scans showed no disease progression.

Case-3 achieved a PR after four cycles of CVP (cyclophosphamide + vincristine + prednisone) chemotherapy with rituximab and an additional four cycles of CHOP with rituximab; subsequent teniposide plus temozolomide was given for four cycles. At the 6-month follow-up visit, the patient exhibited an improved response and the chemotherapy was switched to four cycles of GEMOX (gemcitabine + oxaliplatin). At the 2-year follow-up visit, the patient had stable disease.

Case-4 did not achieve remission after four cycles of EPOCH chemotherapy with rituximab and required a switch to one cycle of Hyper-CVAD (course A, cyclophosphamide + vincristine + dexamethasone; course B, methotrexate + cytarabine) chemotherapy. Additional regional lymph node radiotherapy of the pharyngeal lymphatic ring and upper and middle neck back was performed due to disease progression. The patient had complicating *Acinetobacter baumannii* infection and received gamma globulin infusion. The patient died of respiratory failure at 7-months of follow-up.

## DISCUSSON

NHL is a common complication in AIDS patients, with a prevalence up to 4.3% higher than that in the general population, although HAART can significantly decrease the risk of A-NHL.^13^-^16^ A-NHL more frequently affect adolescents, younger adults, and adults older than 60 years; approximately one-third of A-NHL cases occur within three years after the diagnosis of AIDS, usually with a late stage of the natural AIDS course. The pathogenesis involves multiple factors, including HIV-induced immunosuppression, chronic immune activation stimulated by B lymphocyte antigen, and an imbalance in cytokines.^17^ Moreover, approximately 50% of A-NHL patients have a complicating infection with Epstein-Barr virus or human herpes virus 8.^18^

As in the general population, a variety of NHL subtypes also exist in AIDS patients; however, 95% of A-NHL cases are of B-cell origin and are mainly invasive, including Burkitt lymphoma and diffuse large B-cell lymphoma, as well as rare subtypes of primary central nervous system lymphoma (1−2%), plasmablastic lymphoma, and primary effusion lymphoma (3%).^19^,^20^ The histologic findings among our patients were generally consistent with those of previous reports, mainly including diffuse large B-cell lymphoma and plasmablastic lymphoma.

A-NHL exhibits a highly variable clinical manifestation, which mainly depends on the sites involved. Compared to that in the general population, A-NHL normally has a highly invasive behavior due to disease progression. Almost all A-NHL patients have lymphadenopathy (primarily involving the neck) and frequently suffer from extranodal diseases at sites including the gastrointestinal tract, bone marrow, meninges, and liver, as well as rare occurrences in the lung, heart, orbit, body cavity, kidney, and adrenal gland.^21^ Our patients had head, neck, and trunk lymph node involvement as the initial presentation and also extranodal diseases involving the oral cavity, jawbone, and brain. The use of HAART may decrease the frequency of extranodal involvement in A-NHL patients.^22^ Superficial lymph node disease in A-NHL may be misdiagnosed as septic lymphadenopathy caused by an opportunistic infection and necessitates lymph node biopsy in high-risk patients or those refractory to antimicrobial therapy.

Chemotherapy remains the mainstay of treatment modality for A-NHL; however, potent chemotherapy may have an adverse effect on CD4^+^ cells and subject A-NHL patients to a high risk of opportunistic infection. Before the advent of HAART, low-dose chemotherapy was normally given to A-NHL patients and was associated with a 2-year overall survival of approximately 10%.^23^ The use of HAART has significantly reduced the incidence of A-NHL and improved patients’ prognosis.^24^ Chemotherapy combining pre-emptive HAART resulted in favorable CR and overall survival rates in A-NHL patients, similar to those in HIV-negative NHL patients,^25^,^26^ increasing the 2-year survival up to 75%.^27^ In our case series, the first-line R-CHOP regimen with HAART resulted in a 2-year survival of 60%. Moderate or severe myelosuppression is the major complication after chemotherapy plus HAART. Zidovudine was switched to stavudine in one of our patients due to serious myelosuppression and complicating sepsis. Moreover, our follow-up CD4+ cell count assay results showed a positive association between the CD4+ cell count and A-NHL patients’ survival; for a patient with an increased CD4^+^ cell count, the overall survival was relatively longer although in the presence of late-stage disease and a higher IPI.

The CHOP regimen combined with HAART is the standard chemotherapy for A-NHL cases, mainly including cases of diffuse large B-cell lymphoma,^28^,^29^ and the addition of rituximab has a beneficial effect on high CD20-expressing patients,^26^,^28^ treated mainly with R-CHOP, R-CDE, and R-EPOCH (chemotherapy regimen including Rituxan, etoposide, prednisone, epirubicin, cyclophosphamide, and changchun sheen). No definitive salvage regimen has been established for recurrent or refractory A-NHL. The still debated high-dose chemotherapy and autologous marrow stem cell transplantation may benefit A-NHL patients to an extent similar to that in HIV-negative patients.^30^-^33^ All of our patients received CHOP-based chemotherapy with or without rituximab as the first-line treatment; intensified salvage chemotherapy was given to three patients with a PR or disease progression, which resulted in stable disease in two of three patients.

HAART plays a fundamental role in treatment of A-NHL and improves patients’ tolerance of chemotherapy by promoting immune reconstruction. Powles *et al*.^34^ reported that the combination of HAART and chemotherapy maintained an unchanged HIV RNA load, although the CD4^+^ cell count significantly declined within the first 3 months of chemotherapy and returned to the baseline level 1 month after completion of chemotherapy. It should be kept in mind that A-NHL patients with a CD4^+^ cell count less than 100×10^6^/L must receive antimicrobial prophylaxis against opportunistic infection, especially when rituximab is added.^26^ In our study, case 4 had an extremely low CD4^+^ cell count and died of respiratory failure with complicating grade 4 myelosuppression and serious sepsis.

A-NHL, a less common medical condition encountered in oncologic practice, usually exhibits a highly variable clinical profile and a histologic origin of B cells in the majority of cases. A-NHL patients normally have a poor prognosis due to the late disease stage. Lymph node biopsy needs to be performed in AIDS patients with unexplainable superficial lymph node disease in order to detect A-NHL. Pre-emptive HAART combined with chemotherapy as the first-line treatment with or without molecular targeted therapy can improve A-NHL patients’ survival, whereas the most appropriate salvage treatment remains debatable for A-NHL patients who fail to achieve a CR or who have recurrent or progressing disease. The CD4^+^ cell count may show treatment-related improvement or a reduction throughout HAART and chemotherapy. An improved CD4^+^ cell count is associated with a better clinical outcome in A-NHL patients.
